# An Observational Cohort Study on Delayed-Onset Infections after Mandibular Third-Molar Extractions

**DOI:** 10.1155/2017/1435348

**Published:** 2017-05-21

**Authors:** Giulia Brunello, Marleen De Biagi, Giulia Crepaldi, Fernanda Izaura Rodrigues, Stefano Sivolella

**Affiliations:** ^1^Department of Management and Engineering, University of Padova, Stradella S. Nicola 3, 36100 Vicenza, Italy; ^2^Department of Neurosciences, School of Dentistry, University of Padova, Via Giustiniani 3, 35128 Padova, Italy; ^3^School of Dentistry, Department of Social and Preventive Dentistry, Sao Paulo State University (UNESP), Campus Araçatuba, R. José Bonifàcio 1193, 16015-050 Araçatuba, SP, Brazil

## Abstract

**Objectives:**

The purpose of the present study was to investigate the occurrence and clinical features of delayed-onset infections after mandibular third-molar extractions.

**Method and Materials:**

An observational cohort study was conducted on 179 patients undergoing mandibular third-molar extraction between January 2013 and December 2015, for a total of 217 extractions. Data were recorded at the time of extraction (*T*_0_), on suture removal seven days later (*T*_1_), and 30 days after the extraction, when patients were contacted and asked about their healing process (*T*_2_). The statistical analysis was performed with nonparametric tests. A *p* value lower than 0.05 was considered statistically significant.

**Results:**

Eight delayed-onset infections were recorded, amounting to 3.7% of all extractions. The median time elapsing from the extraction to the delayed-onset infection was 35 days (IQR 28–40; min 24–max 49). Younger age and longer surgical procedures seemed to be more often associated with this complication.

**Conclusion:**

Delayed-onset infections after third-molar extractions are relatively rare postoperative complications characterized by a swelling, usually with a purulent discharge. Patients should be informed of this possibility, which might develop even several weeks after the extraction.

## 1. Introduction 

Lower third-molar extraction is one of the most common procedures carried out by oral and maxillofacial surgeons. The incidence of postoperative complications after mandibular third-molar extractions and the associated risk factors have been amply discussed in the literature [1–6]. Infections are reportedly one of the possible complications after this procedure [1, 5], but few studies have considered a follow-up beyond the first week after surgery, which is usually when patients return for suture removal [3, 4, 7, 8].

Delayed-onset infections (DOI) after mandibular third-molar extractions have been described as a rare complication characterized by swelling, usually with a purulent discharge at the extraction site, developing approximately a month after surgery [3, 7].

The incidence of such DOI reported in the literature ranges between 0.5% and 1.8% [7, 9–12]. The risk factors identified as being associated with this complication are gender (female) and tooth position (mesioangular or vertically tilting, with total mucosa retention, or deep bony impaction). It is not clear whether the ability of the surgeon can influence the occurrence of this complication [7, 8].

In most cases, the treatment of choice involves systemic antibiotics (generally amoxicillin clavulanate) and local antimicrobial mouthwashes (e.g., chlorhexidine 0.2%). When the antibiotic treatment is ineffective, then surgical debridement of the extraction site becomes necessary [13]. The bacteria identified in DOI are* Fusobacterium*,* Prevotella*,* Bacteroides,* and* Peptostreptococcus* [14]. DOI usually occur about thirty days after the extraction, but they may also develop much later on [7, 8, 14].

The purpose of the present study was to analyze the postoperative course after mandibular third-molar extractions in a group of patients followed up for at least a month after surgery. The specific aims of this study were to ascertain the occurrence of DOI after mandibular third-molar removal and to identify any main factors associated with their occurrence.

## 2. Materials and Methods

### 2.1. Study Design

An observational cohort study was conducted on healthy individuals (classified as ASA 1 or 2 according to the Physical Status Classification System) who underwent surgery for the extraction of at least one lower third-molar as outpatients at the Oral Surgery Department of the University of Padua between January 2013 and December 2015.

The study was approved by the local institutional review board (Padua Hospital Ethical Committee, prot. n. 0035161) and it complied with the Helsinki Declaration. All individuals enrolled in the study signed a detailed informed consent form.

### 2.2. Surgical Technique

Patients had an orthopantomogram preoperatively and, if necessary, a computed tomography (CT) scan. All patients were treated by specialists or residents. A lower third-molar was extracted under local anesthesia generally involving an inferior alveolar nerve block associated with a buccal nerve block (2% mepivacaine with 1 : 100,000 epinephrine, Optocain, Molteni Dental, Italy), plus sedation as necessary [15]. All the surgical materials and the surgical field were sterile. An adequate full-thickness flap was raised and, when necessary, ostectomy and tooth sectioning were performed using a straight handpiece with dedicated burs under copious irrigation with sterile saline solution.

Once the tooth had been extracted, the flap was repositioned and sutured (Novosyn 4.0, B. Braun AG, Melsungen, Germany). After surgery, patients were prescribed antibiotics (amoxicillin clavulanate 1 g every 12 hours for 6 days or clarithromycin 250 mg every 12 hours for 6 days), painkillers (paracetamol 1000 mg every 8 hours), and a mouthwash (0.2% chlorhexidine every 12 hours for 3-4 days). They also received standard postoperative recommendations regarding physical therapy, appropriate diet, and the avoidance of smoking.

### 2.3. Data Collection

Clinical data were collected at three different time points: at the time of surgery (*T*_0_); at the time of suture removal one week later (*T*_1_); and thirty days after the extraction (*T*_2_). Beyond *T*_2_, patients were asked to contact the surgeons in the event of any symptoms at the surgical site.

At the baseline (*T*_0_) the surgeon recorded patients' details (gender, age, weight, height, systemic diseases, drug intake, smoking habit, and mouth opening) and features of the tooth to be extracted (stage of root maturation, side of extraction, depth of impaction, angulation, Winter classification, and proximity to the alveolar nerve). If a CT scan had been prescribed before surgery, details of this were also recorded. Patients were asked if any previous pericoronitis had occurred and, in the event of signs of infection at the time of surgery, if they had previously been taking antibiotics.

The following data were collected on the surgical procedure: surgeon's experience (specialist or resident); use of sedation; duration of the surgical procedure; flap design; any ostectomy and tooth sectioning required; and intraoperative complications. Any cortisone (Kenacort 40 mg/mL; Bristol-Myers Squibb, Italy) administered immediately after the surgical procedure was also recorded.

At *T*_1_, any signs of wound dehiscence, swelling at the extraction site, exudate, pus, swollen lymph nodes, pain on palpation, bleeding, or alveolitis were recorded. Patients were given specific information concerning the signs and symptoms associated with infectious and neurological complications. They were asked to contact the surgeon immediately if in any doubt about their postoperative condition in order to schedule a visit and receive appropriate treatment.

At *T*_2_, patients received a follow-up phone call^3^ and were asked if and when any infections had occurred and if and how they were treated (surgery and/or drugs). Patients were asked to contact the surgeon to arrange an appointment in the event of any signs or symptoms involving the extraction site developing after this *T*_2_ phone call. Data concerning patients enrolled for the study who returned to the clinic with a DOI or other complications related to the extraction prior to *T*_2_ were also recorded.

### 2.4. Statistical Analysis

Continuous numerical data were expressed as means and standard deviations (SD) or as medians and interquartile ranges (IQR). Categorical data were compared with Fisher's test. Continuous data were compared with the Mann—Whitney test. No multivariate analysis was conducted on the risk factors for DOI due to the small number of DOI identified. A *p* value of less than 0.05 was considered significant. The statistical analysis was run with the R 3.2.2 language (R Core Team, 2016).

## 3. Results

Data were recorded on 217 lower third-molar extractions performed in 179 patients. Gender and age distributions are shown in Table 1. Eight DOI were identified, amounting to 3.7% of the sample (95% CI 1.6%–7.1%). The median time elapsing from the extraction to the DOI was 35 days (IQR 28–40), with the earliest DOI developing 24 days after surgery and the latest after 49 days.

Table 1 shows the association between patient-related features and DOI events. Patients who developed a DOI were younger, though not significantly so (*p* = 0.06) (Table 1).

Among the features recorded at the time of surgery, only the duration of the surgical procedure differed significantly, being longer for the patients who developed a DOI (*p* = 0.02) (Table 2).

Concerning the Winter classification, the teeth extracted from sites where DOI subsequently developed were characterized as follows: 4 mesioangular, 1 vertical, 1 distoangular, and 2 horizontal. Six of these teeth were fully impacted, the other two semi-impacted (Table 3).

The presence of pus was recorded at *T*_1_ in one of the patients who developed a DOI (*p* = 0.04). None of the other variables considered differed statistically between patients who did or did not develop late infections (Table 4).

As regards the treatment for DOI, antibiotics sufficed in 4 of the 8 cases, while the other 4 required additional surgical procedures.

## 4. Discussion

In the literature the reported incidence of postoperative infections after third-molar removal ranges between 0.9% and 5.8% [1, 3, 4, 16–18]. Many articles focus on postoperative complications, but few studies have investigated the incidence of delayed-onset infections [3, 4, 7, 8]. Two of the latter are retrospective [7, 8], and two [3, 4] are presented as prospective studies, though only Blondeau and Daniel [3] clearly state the method used to collect data and detect any occurrences of DOI. As in the present study, they describe contacting patients by phone four weeks after surgery to obtain information about the onset of any complications.

In a large prospective study on 9,574 patients who underwent a total of 16,127 mandibular third-molar extractions, Osborn et al. [4] reported a 3.4% rate of secondary infections (based on the total number of extractions). The majority of the secondary infections (66%) developed between 15 and 60 days after surgery.

In another prospective study on postoperative complications after lower third-molar extraction, the wound infection rate was reportedly 2.2% at 4 weeks [3].

Christiaens and Reychler [12] conducted a retrospective study on 1,213 maxillary and mandibular third-molar extractions. Infection was the most frequent complication (2.7%), and the secondary infection rate was 1.7% and 3.6% for lower third molars extracted under general and local anesthesia, respectively.

In another retrospective study conducted on 958 extractions, the reported incidence of DOI was 1.5% (95% CI 0.7%–2.2%), but the authors said that a more conservative estimate could reach 2.4% (95% CI 1.2%–3.7%), if only operated patients with a further follow-up after suture removal were taken into account [7].

Delayed-onset infections after third-molar extractions tend to occur about a month after the surgical procedure. In our study, the median time elapsing from surgery to DOI was 35 days (IQR 28–40; min 24–max 49). In the Christiaens and Reychler study [12], 75% of the infections developed 2-3 weeks after the extraction of a third molar. Figueiredo et al. [7] reported similar results, with a mean interval from lower third-molar extraction to DOI of 34.2 days (SD = ±20.3); in another study conducted at the same department [8], the mean interval was 33.4 days (SD = ±3.1); and third, when Figueiredo et al. [14] also investigated the type of bacteria involved in the DOI of 13 consecutive patients, the mean time to occurrence of the infection was 38.7 days. Other studies concerning secondary infections are less clear about the timing of their onset, but it usually comes between 10 and 30 days after surgery [3, 4, 9, 11].

In our sample, patients who developed a DOI were slightly younger than the others (*p* = 0.06), at a mean 18 years of age (IQR 16–25). This finding is consistent with a report from Osborn et al. [4] of a majority of secondary infections occurring in a group of patients between 12 and 24 years old.

All our patients were treated postoperatively with antibiotics and chlorhexidine mouthwash, but this was not enough to prevent the onset of DOI. The effects of these drugs presumably disappear after 3 to 5 weeks, and the changes they induce in the oral flora might favor the development of opportunistic infections [7–9, 11]. In the report from Christiaens and Reychler too [12], the majority of infections occurred 2-3 weeks after the extraction, when patients were no longer under the effect of postoperative antibiotic therapies.

In most of our cases of DOI, the teeth extracted were totally or partially impacted, and ostectomy and tooth sectioning seem to be related to this late complication [3, 4, 7–9, 11]. In terms of the Winter classification, the teeth extracted from sites where a DOI developed in our sample were mesioangular in 4 out of 8 cases. These outcomes confirm other reports, in which mesioangular third molars were more prone to DOI [3, 8]. Figueiredo et al. [8] found that total soft tissue coverage, a lack of distal space, and a mesioangular tilt were significant risk factors for the onset of DOI. They concluded that the reason for this association between the position of the third molar and the onset of infections is probably because the space left empty beneath the soft tissue can be colonized by bacteria across the gingival sulcus. In our sample, there seemed to be a strong correlation between the duration of the surgical procedure and the onset of late infections: a longer procedure was generally associated with fully impacted teeth, which makes their extraction more complicated. Even if it is not clinically evident 7–10 days after the extraction, mucosal healing at the extraction site may be impaired by several factors, including an inappropriate suturing technique in juxtaposing the epithelium on one or both sides of the surgical wound; failure of the epithelium reattachment at the cement-enamel junction of the second molar; food or hematoma trapped under the flap [7–9].

Postoperative complications after third-molar surgery generally seem to be related to a surgeon's inexperience [3, 12, 19], but this aspect was statistically irrelevant in our study. Christiaens and Reychler [12] reported that complications were more frequent when the surgeon was less experienced. Blondeau and Daniel [3] also suggested a correlation between a surgeon's lack of experience and postoperative complications.

## 5. Conclusion 

Delayed-onset infections after third-molar extractions are rare. A younger age of the patient and a longer surgical procedure seem to contribute to the risk of this complication. Surgeons should inform patients of the possibility of a DOI, which is most likely to appear approximately 4 weeks after surgery.

Given the low incidence of DOI after third-molar extractions, a large observational prospective cohort study, with a follow-up beyond one month, would be needed to shed more light on the relationship between this complication and any associated risk factors. The approach to this issue could also be improved by arranging a recall visit 30 days after surgery, but the additional costs would probably not be justified due to the low incidence of DOI.

## Figures and Tables

**Table 1:** Association between patient-related characteristics and delayed-onset infection (DOI).

	Extractions without DOI (*n* = 209)	Extractions with DOI (*n* = 8)	*p* value
Age^*∗*^ (years)	22 (19–28)	18 (16–25)	0.06
BMI^*∗*^	21.6 (19.7–23.8)	20.2 (19.5–21.7)	0.30
Gender M : F	84 : 125	2 : 6	0.48
Presence of systemic diseases	30 (14.4)	2 (25.0)	0.34
Drug intake	29 (13.9)	0	0.60
Smoking habit	66 (31.6)	1 (12.5)	0.44
Mouth opening in mm^*∗*a^	47 (40–50)	45 (5–56)	0.50

Total number of patients 179; total number of extractions 217. Data are expressed as number (%) or ^*∗*^median (IQR). BMI: body mass index. ^a^Data not available in 13 extractions.

**Table 2:** Variables recorded at the time of surgery (*T*_0_).

	Extraction without DOI (*n* = 209)	Extraction with DOI (*n* = 8)	*p* value
Duration of surgical procedure (minutes)^*∗*^	40 (30–55)	50 (48–60)	**0.02**
Sedation	25 (12.0)	0	0.60
Postoperative cortisone	39 (18.7)	1 (12.5)	0.99
Proximity to inferior alveolar nerve	62 (29.8)	2 (25.0)	0.99
Prescribed CT	77 (37.0)	1 (12.5)	0.26
Previous pericoronitis	47 (22.6)	1 (12.5)	0.69
Ongoing infection	10 (4.8)	0	0.99
Ongoing antibiotic therapy	15 (7.2)	0	0.99
Expert surgeon	26 (12.4)	0	0.59
Type of flap			0.86
0 = no flap	13 (6.2)	0	
1 = envelope	151 (72.3)	6 (75.0)	
2 = with vertical buccal-mesial releasing incision	9 (4.3)	0	
3 = other	36 (17.2)	2 (25.0)	
Ostectomy	191 (91.4)	7 (87.5)	0.53
Tooth sectioning	160 (76.6)	8 (100.0)	0.21

Data are expressed as number (%) or ^*∗*^median (IQR).

**Table 3:** Features of the extracted teeth.

	Extraction without DOI (*n* = 209)	Extraction with DOI (*n* = 8)	*p* value
Stage of root maturation^a^			0.31
1 = germ	21 (10.1)	2 (25.0)	
2 = developed more than 1/3 of root	48 (23.1)	2 (25.0)	
3 = developed root	139 (66.8)	4 (50.0)	
Side			0.72
Right	109 (52.2)	5 (62.5)	
Left	100 (47.8)	3 (37.5)	
Tilt^b^			0.88
D distoangular	21 (10.1)	1 (12.5)	
M mesioangular	109 (52.7)	5 (62.5)	
H horizontal	25 (12.1)	1 (12.5)	
V vertical	52 (25.1)	1 (12.5)	
Type of retention			0.12
1 = total	64 (30.6)	5 (62.5)	
2 = partial	103 (49.3)	3 (37.5)	
3 = none	42 (20.1)	0	

Data are expressed as number (%). Data not available in ^a^1 and ^b^2 extractions.

**Table 4:** Clinical features recorded at *T*_1_.

	Extraction without DOI (*n*=209)	Extraction with DOI (*n* = 8)	*p* value
Dehiscence	56 (26.8)	1 (12.5)	0.68
Swelling	50 (23.9)	1 (12.5)	0.68
Exudate	8 (3.8)	1 (12.5)	0.29
Pus	0	1 (12.5)	**0.04**
Lymph node enlargement	22 (10.5)	2 (25.0)	0.22
Pain on palpation	46 (22.0)	2 (25.0)	0.99
Bleeding	5 (2.4)	0	0.99
Alveolitis	7 (3.4)	0	0.99
Trismus	12 (6.2)	1 (12.5)	0.42

Data are expressed as number (%).
